# Idiopathic Juxtafoveolar Retinal Telangiectasis: A Current Review

**DOI:** 10.4103/0974-9233.65501

**Published:** 2010

**Authors:** Sawsan R. Nowilaty, Hanan N. Al-Shamsi, Wajeeha Al-Khars

**Affiliations:** Vitreoretinal Division, King Khaled Eye Specialist Hospital, Riyadh, Kingdom of Saudi Arabia

**Keywords:** Fundus Autofluorescence, Idiopathic Juxtafoveolar Retinal Telangiectasis, Idiopathic Macular Telangiectasia, Laser Photocoagulation, Optical Coherence Tomography, Photodynamic Therapy, Parafoveal Telangiectasis, Subretinal Neovascularization, Vascular Endothelial Growth Factor

## Abstract

Idiopathic juxtafoveolar retinal telangiectasis (IJFT), also known as parafoveal telangiectasis or idiopathic macular telangiectasia, refers to a heterogeneous group of well-recognized clinical entities characterized by telangiectatic alterations of the juxtafoveolar capillary network of one or both eyes, but which differ in appearance, presumed pathogenesis, and management strategies. Classically, three groups of IJFT are identified. Group I is unilateral easily visible telangiectasis occurring predominantly in males, and causing visual loss as a result of macular edema. Group II, the most common, is bilateral occurring in both middle-aged men and women, and presenting with telangiectasis that is more difficult to detect on biomicroscopy, but with characteristic and diagnostic angiographic and optical coherence tomography features. Vision loss is due to retinal atrophy, not exudation, and subretinal neovascularization is common. Group III is very rare characterized predominantly by progressive obliteration of the perifoveal capillary network, occurring usually in association with a medical or neurologic disease. This paper presents a current review of juxtafoveolar retinal telangiectasis, reviewing the classification of these entities and focusing primarily on the two most common types encountered in clinical practice, i.e., groups I and II, describing their clinical features, histopathology, natural history, complications, latest results from imaging modalities and functional studies, differential diagnosis, and treatment modalities.

## INTRODUCTION, TERMINOLOGY AND CLASSIFICATIONS

Idiopathic juxtafoveolar telangiectasis (also known as idiopathic parafoveal, perifoveal or macular telangiectasia or telangiectasis) is a descriptive term for various disease entities presenting with incompetence, ectasia, and/or irregular dilations of the capillary network affecting only the juxtafoveolar region of one or both eyes. These entities are distinguished from more generalized retinal telangiectasis (such as in Coats’ disease) or secondary juxtafoveal retinal telangiectasis due to retinal vein occlusion, diabetes, irradiation, or carotid artery obstruction.

The term *idiopathic juxtafoveolar retinal telangiectasis* (IJFT) was coined by Gass and Oyakawa^1^ in 1982, who proposed the first classification of these entities into four groups based largely on their clinical and fluorescein angiographic (FA) features. In 1993, Gass and Blodi^2^ further updated this classification, by subdividing IJFT into three distinct groups I, II, and III (also known as groups 1, 2, and 3), with two subgroups in each (A and B), based on demographic difference or clinical severity [Table 1]. Each main group had a presumed independent etiology. Group I was congenital and predominantly presenting in males with unilateral telangiectasis and macular edema. Group II was acquired bilateral telangiectasis with atrophy of the fovea. Group III was extremely rare and characterized by progressive obliteration of the perifoveal capillary network. This classification, based on clinical examination and angiographic findings comprised additional staging for IJFT group II into five stages, and proposed observations and pathogenetic mechanisms which continue to be validated today with more modern imaging modalities and functional studies. Despite its complexity, the Gass–Blodi classification is the most commonly used to date.

**Table 1:** Classification of Idiopathic Juxtafoveolar Retinal Telangiectasis.

According to Yannuzzi *et al*^3^→	Aneurysmal Telangiectasia		Perifoveal Telangiectasia			
	I-A:	I-B:	II-A:	II-B:	III-A:	III-B:
According to Gass and Blodi^2^→	Visible & Exudative	Visible, Exudative & Focal	Occult & Nonexudative	Juvenile, occult and familial	Occlusive	Occlusive Associated with Central Nervous System Vasculopathy
Frequency	Second most common	Rare	Most common	Extremely rare	Very rare	Very rare
Gender	Male (90%)	Male	Male=Female		Female	Male=Female
Age (Mean yrs)	15−54 (40)	middle age	35−65 (56)	2 siblings< 12 yrs	40−60	middle age
Congenital/Acquired	Congenital	Congenital	Acquired	Acquired	Acquired	Dominant inheritance
Biomicroscopic Features						
Laterality	Unilateral	Unilateral	Bilateral (asymmetric)	Bilateral	Bilateral	Bilateral
Juxtafoveolar telangiectasis location	Temporal half of macula	Temporal half of macula	Temporal to fovea++++ up to entire perifovea	Temporal to fovea	Perifoveal	Perifoveal
Juxtafoveolar telangiectasis size	≥ 2DD	< 2 clock hours	≈ 1DD around foveola	Small	Variable	Variable
Visual acuity at presentation	≈20/40	≥ 20/25	20/20-20/300	NM	20/25-20/50	variable
Visible macular edema	+++	±	− (unless SRNV)	−	NM	NM
Loss of retinal transparency	+	±	+	+	+	NM
Easily visible microaneurysms/telangiectasis	+++	+	−	−	+	+
Yellow exudates	+	±	−(unless SRNV)	−	Minimal	Minimal
Blunted right-angled venules	−	−	+	−	−	−
Superficial crystalline deposits	−	−	+	−	−	−
ntraretinal pigment plaques/hyperplasia	−	−	+	−	−	−
Fibrous metaplasia	−	−	+	NM	−	−
Subretinal neovascularization/RCA	−	−	+ (in stage 5)	+ (OU)	−	−
Fluorescein Angiographic Features						
Visible telangiectasia	+++	±	±	±	± *	+
Late intraretinal staining	+	+	+	+	+	Minimal
Capillary occlusion	Minimal	−	−	NM	+++ *	+++
Cause of Vision Loss	Macular edema, exudation	No visual loss	Foveal atrophy SRNV	SRNV	Capillary occlusion & obstruction	Capillary occlusion & obstruction
Systemic Associations	None	None	Possible diabetes mellitus	None	Polycythemia Hypoglycemia Gouty arthritis Ulcerative colitis Multiple myeloma Chronic lymphocytic Leukemia	CNS involvement Extra macular telangiectasis

* Resembles sickle cell & radiation retinopathies;

DD - disk diameters; NM - Not mentioned; RCA- Retino-choroidal anastomosis; SRNV - Subretinal neovascularization.

More recently, based on newly recognized clinical, angiographic, and optical coherence tomography (OCT) imaging observations, Yannuzzi **et al**.^3^ proposed a simplified classification of IJFT, essentially a revision and simplification of the Gass–Blodi model. They proposed the term “idiopathic macular telangiectasia” with two distinct types: Type 1 or “aneurysmal telangiectasia” equivalent to IJFT group I (A and B combined), which is the second most common form of IJFT; and type 2 or "perifoveal telangiectasia" equivalent to IJFT group IIA, the most common type of IJFT. The remaining types described by Gass and Blodi (group IIB and groups IIIA and B) were omitted from Yannuzzi’s classification because of their rarity. Yannuzzi *et al*.^3^ furthermore simplified the five stages of group IIA proposed by Gass and Blodi into two distinct stages which have clinical, therapeutic, and prognostic relevance: nonproliferative and proliferative stage [Table 2]. Other recent classifications based on OCT phenotypic findings were proposed as well (detailed below). All these classifications nevertheless concur with the already established observations and classifications by Gass and associates.^1^,^2^

**Table 2:** Clinical stages of idiopathic juxtafoveolar retinal telangiectasis group IIA (idiopathic macular telangiectasia type 2)

Stage		Prominent feature	Visual symptoms	Biomicroscopy	Fluorescein angiography
Yannuzzi *et al*.^3^	Gass and Blodi^2^				
Nonproliferative perifoveal telangiectasia	1	Occult vascular abnormalities	Asymptomatic	Slight parafoveal graying (may be difficult to detect)	Minimal or no evidence of capillary dilation and late retinal staining
	2	No clinically visible telangiectasis	Asymptomatic or mild visual disturbances	Mild loss of parafoveolar retinal transparency/no visible telangiectatic vessels/superficial refractile crystals possible	Early staining of capillary walls in outer retinal network/diffuse late staining
	3	Prominent dilated right-angled retinal venules	Metamorphopsia, mild scotoma	Parafoveal right-angled venules draining telangiectasis/visible capillary dilation	Capillary dilation and leakage beneath right-angled venules causing late retinal staining/no pooling of fluid
	4	Retinal pigment hyperplasia extending into the retina	Progressive visual decline	Retinal pigment epithelial hyperplasia or clumps around right-angled venules	Capillary dilation and leakage beneath right-angled venules causing late retinal staining/no pooling of fluid/blocked fluorescence in areas of pigment
Proliferative perifoveal telangiectasia	5	Subretinal neovascularization	Rapid and severe visual loss	Subretinal exudation and hemorrhage/ retinochoroidal anastomosis	Similar to classic neovascularization + features of Stages 3−4

In an attempt for simplicity and consistency, the following review of idiopathic juxtafoveolar telangiectasis will use the original terminology/classification proposed by Gass and Blodi and will focus on the two most common subtypes of IJFT, namely IJFT group I (equivalent to aneurysmal telangiectasia) and IJFT group IIA (equivalent to perifoveal telangiectasia) reviewing the clinical features, histopathology, natural history and complications, latest results from imaging modalities and functional studies, the differential diagnosis, and treatment modalities.

## GROUP I: VISIBLE AND EXUDATIVE IDIOPATHIC JUXTAFOVEOLAR RETINAL TELANGIECTASIS(“Idiopathic Macular Telangiectasia Type 1” or “Aneurysmal Telangiectasia”)

### Clinical, angiographic, and OCT features

**Figure 1:**
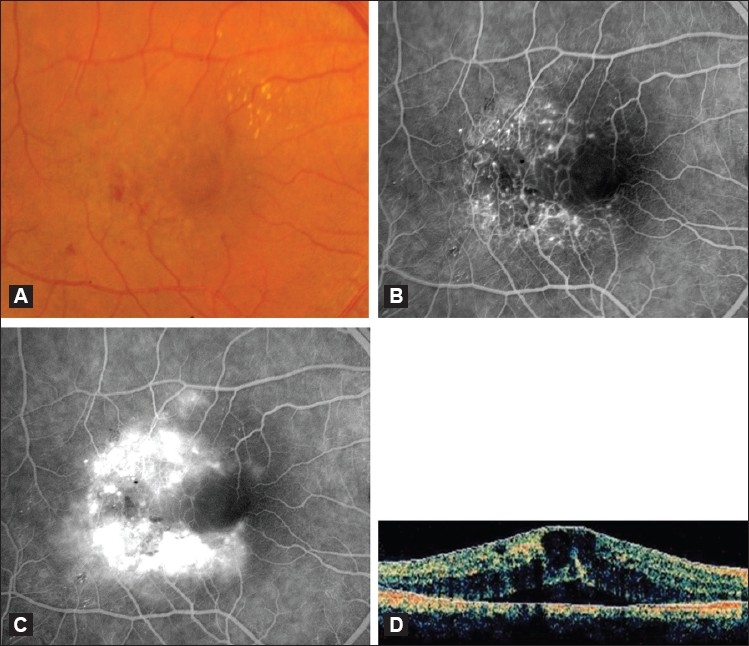
(A) Color fundus photograph of an eye with IJFT I. Note the visible retinal telangiectasis, aneurysmal dilations temporal to the fovea, and the characteristic lipid deposition. (B and C) Corresponding early (B) and late (C) fluorescein angiogram showing easily visible telangiectatic vessels and aneurysmal capillary dilations straddling the horizontal raphe causing late leakage. (D) Optical coherence tomography scan of an eye with IJFT I showing increased central retinal thickness, intraretinal fluid-filled spaces, and subretinal fluid

This congenital or developmental form of IJFT occurs predominantly in males and is typically unilateral (97% of cases).^2^,^3^ Although the onset of symptoms can occur at any age, the mean age at presentation is 40 years. On biomicroscopy, prominent easily visible telangiectatic retinal capillaries, with variable-sized aneurysmal dilations are a consistent hallmark of this type of IJFT.^2^,^3^ The telangiectasis usually involves a two-disc diameter area or greater temporal to the fovea [Figure 1A].^1^–^5^ Macular edema and lipid deposition of variable amount is a characteristic feature. Of note, no blunted right-angled venules, superficial vitreoretinal interface crystalline deposits, plaques of pigment epithelial hyperplasia, intraretinal pigment migration or subretinal neovascularization are seen in this type of IJFT.^2^,^3^

On stereoscopic FA, the telangiectatic vessels are easily visible straddling the horizontal raphe and filling promptly in both the superficial and deep juxtafoveolar capillary plexus [Figure 1B and C].^2^ Minimal nonperfusion or capillary ischemia exists sometimes and is easily visible on FA.^1^–^3^ Central cystic or noncystic macular edema is evident angiographically as late intraretinal staining^2^,^3^ and confirmed with OCT in all cases,^3^ the latter characteristically demonstrating increased central retinal thickness and fluid-filled spaces.^3^,^6^,^7^ In some eyes, the edema extends beneath the retina to produce shallow detachments of the macula visible only with OCT [Figure 1D].^3^ On ultra-high resolution OCT (UHR-OCT), characteristic abnormally large intraretinal blood vessels located near the fovea and deep in the outer nuclear layer can be visualized.^7^ Müller cells bodies span the separation between the outer nuclear layer and outer plexiform layer. Of important note however, the outer retina (i.e., outer nuclear layer, external limiting membrane, and inner segment/outer segment junction), remains uninvolved and juxtaposed to the retinal pigment epithelium (RPE).^7^

Macular edema and exudation are the main cause of visual loss in these patients.^1^–^4^ However, the amount of exudation, cystoid macular edema, and subsequent visual acuity (VA) loss is variable^1^,^2^ The median VA at presentation is 20/40 in Gass’s series.^2^ Some patients retain excellent VA for years without treatment. The vascular malformations may function normally for years and then progress to a pathological state later in life.^1^,^2^ In some cases, spontaneous resolution may occur.^1^,^2^ If progressive visual loss occurs however, treatment with laser photocoagulation may be effective in reducing the exudation and improving or stabilizing vision.^1^,^2^,^8^

In addition to the juxtafoveal vascular lesions, which are essential for the diagnosis, this group of IJFT may also develop focal vascular changes in the mid-peripheral fundus and even in the more anterior fundus.^1^,^3^,^9^ In fact Gass *et al*. believed that this group of IJFT may be a localized form or part of the spectrum of congenital retinal telangiectasis or Coats’ disease, identical to what Leber had previously described as miliary aneurysms of the retina.^1^,^2^,^5^,^8^,^10^ Similarly, Yannuzzi *et al*. consider aneurysmal telangiectasia to be a form of Coats’ disease that is found in the macula.^3^ On histopathology, the findings of IJFT I are consistent with the clinical observations of dilation of capillaries, aneurysms, leakage, and minimal nonperfusion.^11^

We must mention that Gass and Blodi identified a separate subgroup of IJFT I named group IB carrying identical features described above but confined to two-clock hours or less in the juxtafoveolar areas with much better VA.^2^ Yannuzzi *et al*. considered that two-clock hours progress to more extensive disease with time and therefore groups IA and B should be merged under the same umbrella of “aneurysmal telangiectasia.”^3^

### Differential diagnosis

When IJFT I is suspected, it must be differentiated from secondary telangiectasis caused by retinal vascular diseases such as retinal venous occlusions, diabetic retinopathy, radiation retinopathy, sickle cell maculopathy, inflammatory retinopathy/Irvine–Gass syndrome, ocular ischemic syndrome/carotid artery obstruction, hypertensive retinopathy, polycythemia vera retinopathy, and localized retinal capillary hemangioma. In addition, IJFT I should be clearly differentiated from dilated perifoveal capillaries with evidence of vitreous cellular infiltration secondary to acquired inflammatory disease or tapetoretinal dystrophy, or from Coats’ disease which is defined by extensive peripheral retinal telangiectasis, exudative retinal detachment, relatively young age of onset, and male predilection.^5^,^8^ Less commonly, macular telangiectasis has been described in association with fascioscapulohumeral muscular dystrophy, incontinentia pigmenti, and familial exudative vitreoretinopathy with posterior pole involvement.

### Treatment

Treatment options for IJFT I include laser photocoagulation, intravitreal injections of steroids, or anti-vascular endothelial growth factor (VEGF) agents. Photocoagulation was recommended by Gass and remains to date the mainstay of treatment. It seems to be successful in causing resolution of exudation and VA improvement or stabilization in selected patients.^1^,^2^ Photocoagulation should be used sparingly to reduce the chance of producing a symptomatic paracentral scotoma and metamorphopsia. Small burns (100–200 μm) of moderate intensity in a grid-pattern and on multiple occasions, if necessary, are recommended. It is unnecessary to destroy every dilated capillary, and, particularly during the initial session of photocoagulation, those on the edge of the capillary-free zone should be avoided.^2^

Intravitreal injections of triamcinolone acetonide (IVTA) which have proved to be beneficial in the treatment of macular edema by their anti-inflammatory effect, their downregulation of VEGF production, and stabilization of the blood retinal barrier,^12^,^13^ were reported anecdotally in the management of IJFT I. In two case reports, IVTA of 4 mg allowed a transitory reduction of retinal edema, with variable or no increase in VA. As expected with all IVTA injections, the edema recurred within 3–6 months, and no permanent improvement could be shown.^14^,^15^ In general, the effect of IVTA is short-lived and complications, mainly increased intraocular pressure and cataract, limit its use.

Indocyanine green angiography-guided laser photocoagulation directed at the leaky microaneurysms and vessels combined with sub-Tenon’s capsule injection of triamcinolone acetonide has also been reported in a limited number of patients with IJFT I with improvement or stabilization of vision after a mean follow-up of 10 months.^16^ Further studies are needed to assess the efficacy of this treatment modality.

Recently, intravitreal injections of anti-VEGF agents, namely bevacizumab, a humanized monoclonal antibody targeted against pro-angiogenic, circulatory VEGF, and ranibizumab, a FDA-approved monoclonal antibody fragment that targets all VEGF-A isoforms, have shown improved visual outcome and reduced leakage in macular edema form diabetes and retinal venous occlusions. In one reported patient with IJFT I, a single intravitreal bevacizumab injection resulted in a marked increase in VA from 20/50 to 20/20, with significant and sustained decrease in both leakage on FA and cystoid macular edema on OCT up to 12 months.^17^ It is likely that patients with IJFT I with pronounced macular edema from leaky telangiectasis may benefit functionally and morphologically from intravitreal anti-VEGF injections, but this warrants further studies.

Today, laser photocoagulation remains mostly effective, but the optimal treatment of IJFT I is questioned, and larger series comparing different treatment modalities seem warranted. The rarity of the disease however, makes it difficult to assess in a controlled randomized manner.

## GROUP IIA: OCCULT AND NONEXUDATIVE IDIOPATHIC JUXTAFOVEOLAR RETINAL Telangiectasis(“Idiopathic Macular Telangiectasia Type 2“ or “Perifoveal Telangiectasia”)

### Clinical features and natural history

This is the most common type of IJFT, and differs completely from IJFT I. It is acquired, not congenital. Affected patients are middle-aged or older (mean 55 years).^2^,^3^ Males and females are affected equally. This disorder is bilateral, but may be asymmetric appearing as unilateral in its early stages.^2^ Similarly, patients may have visual loss in only one eye.

The natural course of IJFT IIA has been subdivided by Gass and Blodi into five stages.^2^ Although this staging has been simplified recently, the main observations remain identical [Table 2].^3^

**Figure 2:**
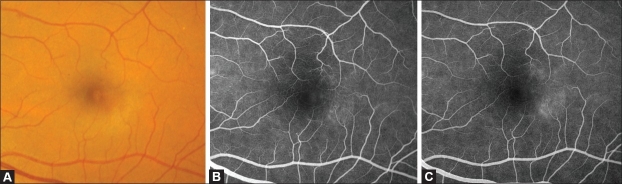
(A) Color fundus photograph of an eye with JFT IIA Stage 1 showing a loss of transparency of the temporal parafoveolar retina. (B and C) Corresponding fluorescein angiogram showing early discrete staining of the temporal parafoveolar capillaries (B), followed by late retinal staining (C). Note the absence of clearly visible telangiectasis

In Stage 1, patients are generally asymptomatic. A slight loss of the retinal transparency, typically grayish and usually in the temporal juxtafoveolar area, where this condition most often starts, may be the only biomicroscopic clue to the presence of the telangiectasis [Figure 2A].^1^,^2^ Fluorescein angiography is required for detection and shows no evidence of capillary dilation, but mild late retinal staining of the outer juxtafoveolar retina surrounding part or all the foveolar border but sparing the foveola itself [Figure 2B and C].^2^,^5^,^8^

**Figure 3:**
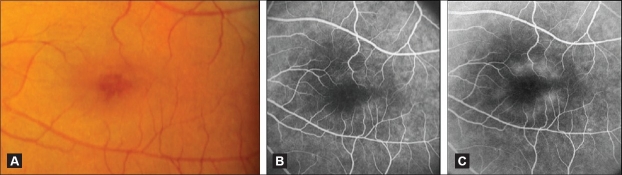
(A) Color fundus photograph of an eye with JFT IIA Stage 2 showing a mild parafoveolar retinal graying in a ring configuration that spares the foveal center. The latter appears darker and thinner. Note the absence of visible telangiectasis. (B and C) Fluorescein angiogram of the same eye demonstrating early hyperfluorescence corresponding topographically to the retinal graying (B), followed by diffuse retinal staining in the late phase (C). Note the central foveal sparing

In Stage 2, patients may be asymptomatic or have minimal disturbances in central vision such as blurred vision, metamorphopsia, or paracentral positive scotoma.^1^ A slight graying of the parafoveolar retina approximately one disc diameter in size, confined temporally or forming a partial or complete horizontal oval around the foveal center exists.^1^–^3^,^18^ The foveal center is spared and may appear thinned [Figure 3A].^2^,^3^ The telangiectasis is minimally or not visible, hence the term “occult” as opposed to the easily visible telangiectasis of IJFT I.^2^,^3^ Superficial crystals may be seen.^1^,^2^,^5^,^8^,^19^ There is little or no thickening.^2^,^3^,^5^,^8^,^20^ Stereoscopic FA demonstrates early rapid staining of the thickened walls of the outer capillary network, mostly temporally, followed by diffuse late staining primarily in the middle and outer retina [Figure 3B and C].^2^,^3^,^5^,^8^,^20^,^21^

**Figure 4:**
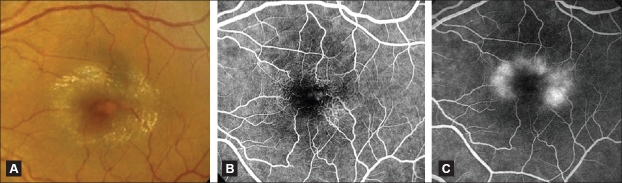
(A) Color fundus photograph of an eye with IJFT IIA Stage 3 showing a grayish ring around the foveal center with numerous superficial retinal crystals and a slightly dilated right-angled venule temporally. (B and C) Corresponding fluorescein angiogram showing in the early phase clearly visible dilation and telangiectasis of the perifoveolar capillary network beneath the right-angled venule (B). These capillaries cause late intraretinal staining (C)

In Stage 3, patients may experience decreased vision, which is slow in onset and progression. Paracentral vertically oriented slightly dilated right-angled venules draining the telangiectatic area are evident biomicroscopically [Figure 4A].^2^ These vessels have been recently attributed both a venular and arteriolar origin that leads to a network of proliferating vessels in the deep retinal layers.^3^ These right-angled vessels typically develop temporally. Prominent dilation of the capillaries may be seen clinically. The foveolar depression may simulate macular hole. Stereo FA often shows unusual capillary dilation and permeability change in the outer retina beneath the right-angled vessels causing the retinal staining.^1^–^3^ However, there is no fluid causing ballooning or cystic spaces in the outer plexiform layer (as occurs in eyes with IJFT I) [Figure 4B and C].

**Figure 5:**
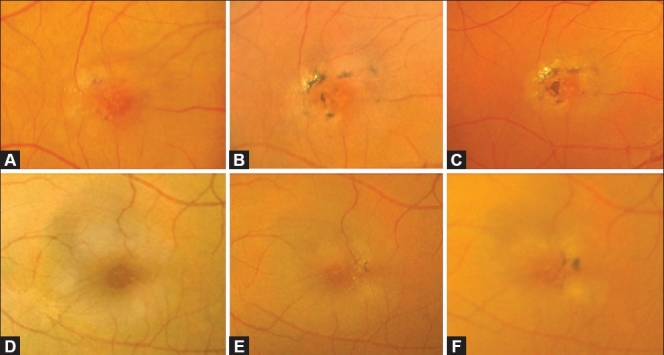
Serial color fundus pictures of the right eye (A-C) and left eye (D-F) of a patient with JFT IIA. (A) Early Stage 4 depicting intraretinal pigment epithelial deposition in the vicinity of the temporal right-angled venule. (B and C) Over a period of 5 years, a progressive increase in the number and size of the intraretinal pigment clumps was observed. The clumps are located preferentially close to the nearby parafoveal vessels. Note the increasing superficial refractile crystals over time, and the central foveal atrophy which is more clearly seen in (C). In the left eye, which was at an earlier stage (Stage 2 in D) similarly showed increasing intraretinal pigment epithlelial migration over time (Stage 4 in E and F). This example illustrates that IJFT II can be asymmetric. Note how the foveal depression simulates a macular hole in this eye (D)

**Figure 6:**
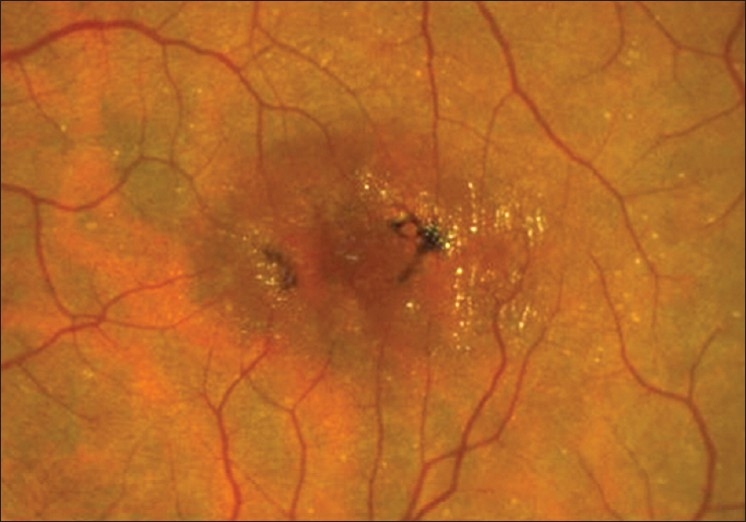
Color fundus photograph of an eye with IJFT IIA Stage 4 showing stellate intraretinal pigment epithelial plaques and refractile retinal crystals

In Stage 4, as a result of RPE migration into the retina along the course of the right-angled vessels, one or more loci of black retinal pigmented epithelial hyperplasia or clumps may be seen around the parafoveolar right-angled vessels [Figure 5]. In some cases, the pigment extends into the inner retina and forms an irregular or stellate plaque enveloping the right angled-vessel [Figure 6].^2^,^20^ Some patients develop a pseudovitelliform lesion within the fovea.^2^,^20^

**Figure 7:**
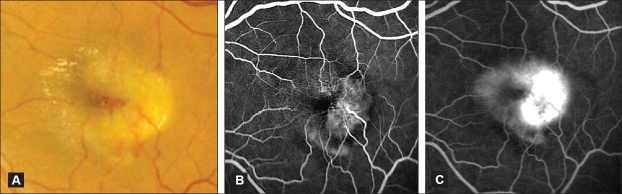
(A) Color fundus photograph of an eye with IJFT IIA Stage 5 (proliferative stage) showing temporal parafoveal retinal elevation with subretinal fluid, mild subretinal lipid exudation, and subretinal blood characteristic of the onset of subretinal neovascularization. Note the superficial refractile crystals nasally. (B and C) Corresponding fluorescein angiogram featuring subretinal neovascualirization, temporal to the foveal center, that is rapidly hyperfluorescent in the early stage (B) increasing in fluorescence and leaking intensely in the late phase (C). Note that the capillary telangiectasis is also easily visible at this stage of the disease (nasally), demonstrating early capillary wall staining and late intraretinal staining that differs in character form the more intense late hyperfluorescent leakage of the subretinal neovascularization

**Figure 8:**
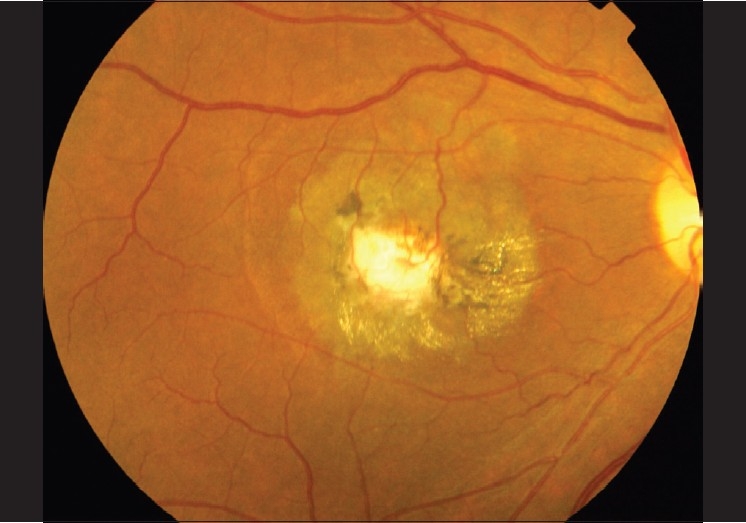
Color fundus photograph of IJFT IIA late Stage 5 that did not receive treatment showing fibrovascular tissue. Note the retinal venules dipping into the fibrous tissue. The superficial retinal crystals are still present

Stage 5 is marked by the onset of SRNV which occurs as a result of retinal capillary remodeling, proliferation, and invasion of the outer retina which has progressively atrophied.^2^,^3^,^20^–^22^ A subretinal network, often with a clearly discernible retino-retinal anastomosis (RRA) or retinal-subretinal anastomosis is evident.^3^ Retino-choroidal anastomosis may exist.^1^ The SRNV usually occurs temporally, often in the vicinity of the intraretinal pigment epithelial migration^1^,^2^ or unrelated to the latter.^3^ Rapid visual decline ensues as the SRNV causes exudation, neurosensory elevation, intra and subretinal hemorrhage, and fibrovascular proliferation. These features are easily evident clinically and angiographically [Figure 7A, B, and C]. On FA, the SRNV has angiographic features similar to classic neovascularization demonstrating early hyperfluorescence which increases and leaks in the late phases of the angiogram.^8^,^23^ However, it is not associated with RPE detachment and its final size is generally smaller compared to classic choroidal neovascularization in AMD and visual acuity does no deteriorate much further.^1^–^3^,^23^ The fibrovascular tissues tend to remodel over time, leading to retinal vascular distortion and dragging of neighboring venules and arterioles into the tissue itself [Figure 8].^23^

At any stage (Stages 2–5), tiny golden crystals may be seen near the retinal surface often anterior to the retinal vessels over the area of telangiectasis. They are characteristic although an inconsistent feature [Figures 4–8].^2^,^3^ According to Yannuzzi *et al*., the intraretinal and subretinal pigmented plaques may also occur variably throughout the course of the disease and may persist without visual consequence for years.^3^

Three key and distinguishing features of IJFT IIA are:

1. The absence of prominent aneurysms or hemorrhage^2^,^3^;
2. The absence of cystic macular edema or lipid exudation (unless SRNV has developed). The loss of retinal transparency and fluorescein staining are primarily caused by intracellular edema (contrary to the extracellular fluid causing CME and lipid exudation in IJFT I)^2^; and
3. The presence of foveolar atrophy, best seen with OCT which can simulate a lamellar macular hole. Foveal atrophy is the primary cause of the slow progressive visual loss occurring over years in these patients (to 20/200 or worse), distinguishable from the rapid and severe visual loss that may occur with the advent of SRNV and fibrosis.^2^,^3^,^20^

As mentioned, the five stages were simplified into two distinct stages that have clinical, prognostic, and therapeutic implications: nonproliferative stage (Stages 1–4), characterized by telangiectasis and foveal atrophy without SRNV, and proliferative stage (Stage 5 of Gass and Blodi) defined by the advent of SRNV and fibrosis [Table 2].^3^

### Histopathology

The histopathological features of IJFT IIA have been described. In a landmark histopathological report, Green *et al*.^24^ did not find telangiectasis of the retinal vessels but, instead, thickening of retinal capillaries from marked proliferation of the basement membrane in a multilayered configuration, and narrowing of the caliber of the lumen. Degeneration of pericytes, and occasionally endothelial cells, was also described, not only in the affected juxtafoveal area, but also to a lesser degree throughout the retina. Cellular debris from degenerated endothelial cells and pericytes, and multimembranous lamellar lipid were entrapped between layers of capillary basement membrane. Localized endothelial defects in the temporal parafoveal area were seen, and the authors postulated that these were the sites at which fluorescein diffused into walls and became trapped by the thickened capillary wall. Intracellular and extracellular edema, especially prominent in the inner retinal layers was observed. Retinal capillary proliferation into the outer retina as far as the photoreceptor layer was also noted.^24^

Another histopathological report of a more advanced case with SRNV corroborated these findings and demonstrated in addition (1) dilation and proliferation of retinal capillaries into the outer retinal layers and into the subretinal space with retinochoroidal vascular anastomosis and (2) intraretinal migration of the RPE along the course of the telangiectatic vessels.^25^

All these histological features corroborate the clinical findings described by Gass *et al*., and although they suggest that the juxtafoveal retinal capillaries are the primary tissues involved in this condition,^21^,^23^,^24^ they also support what Gass has postulated, i.e., that capillary *dilation* and “telangiectasis” does not occur until later in the process.^26^

### Pathophysiology

The precise pathogenesis of IJFT IIA is controversial. In their earlier report and based on the angiographic findings, Gass and associates initially proposed a primary role of the retinal capillaries.^1^ The altered capillary wall associated with metabolic alterations and increased endothelial permeability, incite chronic nutritional damage to the retina, particularly the Müller cells.^27^ Further changes in the outer capillary bed would cause an alteration in the pattern of venous outflow and the formation of right-angled venules. The nutritional deprivation of the middle retinal layers would lead to degeneration and atrophy of the outer retina including the photoreceptors which would cause the gradual visual loss and permit subretinal and intraretinal pigment epithelial migration and proliferation to form black plaques in the vicinity of the right-angled venules. Photoreceptor atrophy would also permit the proliferating capillaries to invade the subretinal space causing SRNV and the latter may develop anastomosis with the choroidal vessels.^2^,^21^,^23^ In later years however, Gass commented that IJFT IIA “is not primarily a leaky retinal blood vessel disease,” but rather “the primary abnormality may reside in one or both of the parafoveolar retinal neural or Müller cells” since he observed that telangiectatic capillary dilation does not occur until later in the process, and the loss of central vision is due to photoreceptors atrophy in the absence of macular edema.^26^,^27^

Although, the clinical pathologic report by Green *et al*.^24^ showed intracellular and intercellular edema primarily in the inner retinal layers as well as capillary endothelial cell degeneration and regeneration, the unusual capillary endothelial abnormalities observed could, in fact, be secondary to Müller cell dysfunction. Müller cells in the retina, like astrocytes in the brain, are critical to proper function of the retinal capillary endothelium and the health of the surrounding neurons.^28^–^31^ A primary degeneration or dysfunction of the parafoveolar Müller cells, could lead in this scenario to (1) retinal endothelial cell degeneration as suggested by Gass and Yannuzzi,^3^,^27^ or accelerated rate of cell death and replacement as suggested by Green^24^ as a possible triggering mechanism for proliferation of retinal capillaries; and (2) retinal thinning, accompanied later by a breakdown of the blood–retinal barrier at the parafoveolar retinal capillaries, causing a superimposed limited edema. The precise mechanism for the retinal capillary proliferation, however, remains unknown.

Superficial crystalline deposits are a common finding in IJFT IIA and are thought to represent the remnants of degenerated Müller cells because of their location near the internal limiting membrane (ILM).^2^ The process of subretinal proliferation is similar to the neovascular form of age-related macular degeneration known as retinal angiomatous proliferation (RAP).^32^ Both IJFT IIA and RAP can be associated with retino-retinal anastomoses that may extend beneath the retina when SRNV occurs^33^,^34^ and plaques of subretinal pigmentation.^35^ The latter are hypothesized to represent reactive hyperplasia of the RPE induced by extension of the retinal vascular proliferation posteriorly toward it.^3^

### Epidemiology/Associations

IJFT IIA is the most common type of IJFT. In the largest two series, the case frequency ratio of IJFT I/IJFT II/IJFT III was 1.0/3.1/0.2 cases, respectively.^2^,^3^ The prevalence of IJFT IIA, however, is unknown. In an Australian population, the prevalence estimates of IJFT IIA Stages 2–3 or higher were 5–23 cases per 100,000 people aged above 47 years. The prevalence is probably higher because early cases, usually asymptomatic, go undetected.^36^

IJFT IIA has been reported in monozygotic twins^37^–^39^ as well as in siblings and families,^2^,^6^,^40^–^42^ suggesting that the condition may have a genetic component. A defective ataxia telangiectasia (ATM) gene has been suggested.^43^ No gene has been identified to date.

Although a link between IJFT IIA and diabetic retinopathy on the basis of histological similarities and a high incidence of abnormal glucose tolerance tests in these patients^41^,^44^,^45^ was suggested, long-term follow-up studies have failed to prove a definitive link to diabetes in a majority of these patients.^2^,^43^ In one series, 19.2% of patients with IJFT IIA had diabetes mellitus with no clinical diabetic retinopathy, and this incidence of diabetes was considered similar to that reported in the general population of the same age.^3^

### Morphologic alterations beyond funduscopy and angiography

The initial characterizations of IJFT IIA relied on clinical observation and FA.^1^,^2^ Newer and noninvasive imaging methods used for studying retinal anatomy and function, are enhancing the morphologic characterization of this disease and providing insights into its pathogenesis.

Several studies of IJFT IIA using OCT, a noninvasive technique that provides cross-sectional *in vivo* imaging of the retina either with an axial resolution of ~7–10 μm for commercially available systems^3^,^6^,^7^,^46^–^54^ or 3 μm for the UHR–OCT,^7^ have provided useful information that may potentially improve the understanding of the pathogenesis and causes of visual loss as well as enhance the diagnosis and treatment of this disease.

**Figure 9:**
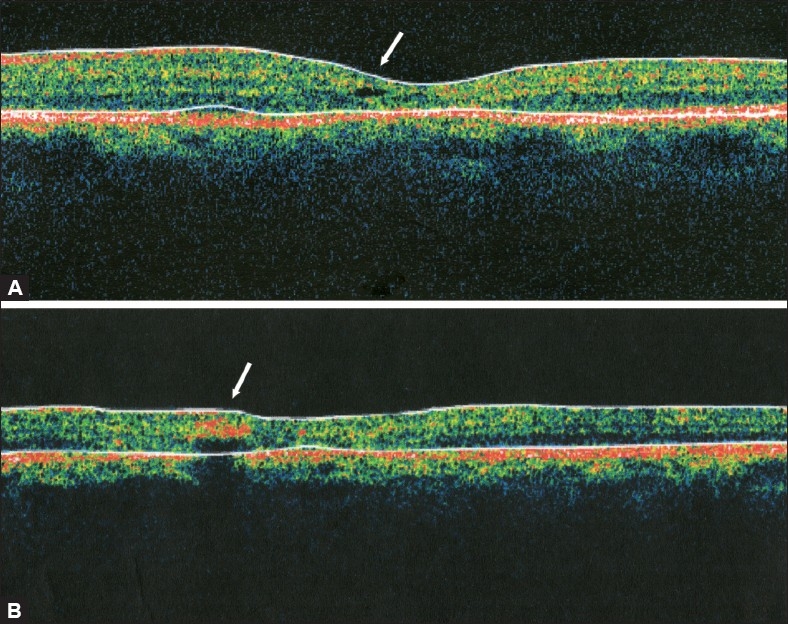
(A) Optical coherence tomogrpahy scan at the fovea demonstrating a small inner lamellar retinal cyst “cystoid” (arrow) typical of IJFT IIA. Note the absence of retinal thickening or fluid-filled spaces. (B) Optical coherence tomogrpahy scan demonstrating an intraretinal hyper-reflective area (arrow) that corresponds to an intraretinal pigment epithelial plaque which causes posterior shadowing

The following OCT features were described in IJFT IIA:

1. Retinal thickness is variable and does not correlate with the degree of leakage seen on FA.^6^,^7^,^46^,^47^,^49^–^52^ Central foveolar thickening is consistently absent,^47^ and intraretinal edema is either absent^6^ or minimal^3^,^7^,^47^,^49^–^52^ superimposing over a fovea of normal or reduced thickness.^47^,^53^–^54^
2. Thinning and disruption of photoreceptor layer, more readily visualized by UHR–OCT, is common,^7^,^47^,^48^ confirming that foveal atrophy is the primary cause for reduced vision. This disruption was found to correlate with VA in 63% of eyes^7^ and increased with advancing disease.^47^,^48^ This finding may be of importance in guiding treatment decisions.
3. Cyst-like structures in the foveola and inner retinal layers are very common (50–100% of eyes with Stage 3 or higher) [Figure 9A]. 3,6,7,47–49,51,52 They are referred to as “cystoids,” have variable size and are not seen clinically or on FA.^48^ Because of the consistent absence of associated cystoid macular edema^6^,^47^ or petaloid pooling on FA, these cysts are unlikely a result of exudation, but rather a progressive retinal tissue loss.^6^,^47^,^49^ At the foveola, the inner lamellar cyst appears as a loss of tissue^3^,^7^ with the ILM spanning across it and draping over it.^7^ This unique feature, the ILM drape, may be specific to IJFT IIA.^7^ This feature is what was previously described as a lamellar retinal hole by Gass who did not have the advantage of OCT imaging.^2^,^5^ Full-thickness macular holes are uncommon in IJFT IIA but have been reported.^55^,^56^ In most eyes with cysts VA is decreased (20/40 to 20/70),^6^ and cyst enlargement has been reported with disease progression and more pronounced visual loss.^3^,^6^
4. Blunting of the foveal pit is common to all stages.^48^ Foveal flattening or thinning is encountered with more advanced disease.^6^
5. Intraretinal neovascularization near the foveola, seen as highly reflective dots in the inner and outer nuclear layers^48^ is found in 21% of eyes.^7^
6. Central intraretinal hyper-reflective lesions that cause nonspecific posterior shadowing and correspond to hyperpigmented RPE plaques are observed [Figure 9B].^3^,^5^,^57^ The crystalline deposits are generally too small to induce photoreflectance.^3^

All of the changes mentioned above (foveal cyst, intraretinal RPE hyperplasia, foveal atrophy, and absence of edema) are consistent with the hypothesis of progressive retinal tissue loss, possibly due to Müller cells degeneration.^26^,^47^ The latter could lead to alterations in the parafoveolar retinal capillaries, to ILM separation, tissue loss, and cyst formation in the fovea.^3^,^6^ Parallel to these changes, or later in the course of the disease, reduction of foveal thickness from resolution of foveal cyst or photoreceptor atrophy occurs.^6^,^47^

The new findings on OCT, such as intraretinal cysts and retinal thickening superimposed on an atrophic neurosensory retina, represent important steps in the evolution of the disease.^3^,^6^,^7^,^47^,^49^–^52^ This has prompted some authors to propose new, OCT-based, staging of IJFT IIA. Cohen 47 proposed the following stages for nonproliferative IJFT IIA; Stage 1: Normal anatomy; Stage 2: No cysts are observed. Photoreceptor disruption may be seen in 18% of eyes; Stage 3: One or more foveal cysts appear. Photoreceptor disruption and outer retinal atrophy are observed in 86% of eyes; Stage 4: A hyper-reflective plaque and photoreceptor disruption and outer retinal atrophy are constant. Foveal cyst(s) are seen in most eyes (64%). Another staging was described by Sanchez.^48^ Stage 1: Inner retinal highly reflective dots corresponding to microvessels on FA in (62.5% of eyes); Stage 2: Intraretinal cysts (81.8%), highly reflective dots (90.9%), and absent retinal thickening; Stage 3: Similar reflectivity of the outer and inner retina with thickening or disruption of RPE/choriocapillaris complex (81.2%); Stage 4: An intraretinal highly reflective area nasal or temporal to the fovea corresponding to RPE proliferation (100%); Stage 5: Fusiform thickening and duplication of the highly reflective RPE/choriocapillaris complex corresponding to choroidal neovascularization (100%). The usefulness of OCT-based staging in clinical practice is yet to be determined. Nevertheless, OCT imaging is offering a new opportunity to understand the pathogenesis and natural history of this perplexing retinal disease.

In recent years, confocal reflectance imaging using the confocal scanning laser ophthalmoscope (cSLO; HRA2, Heidelberg Engineering, Heidelberg, Germany), has emerged as a very sensitive and noninvasive method for the diagnosis of IJFT IIA, monitoring its progression as well differentiating it from other conditions.^58^,^59^ Confocal blue reflectance (CBR) imaging (at 488 nm) is particularly helpful in the diagnosis of the early stages of IJFT IIA which are the most difficult to detect clinically, disclosing a well-defined generally oval parafoveal area of increased reflectance that corresponds to, but is slightly larger than, the area of leakage in late-phase angiography.^46^,^58^,^59^ Confocal infrared reflectance (at 820 nm), on the other hand, is helpful in monitoring progression, showing in the early stages, a uniform increased reflectance corresponding to the area of leakage on angiography, and in late stages with pigment clumping or SRNV, a decreased reflectance in the area of leakage.^58^ Interestingly, the presence in the early stages of an increased CBR area that is larger than the area of angiographic leakage supports the assumption that the angiographically visible vascular alterations could be a secondary phenomenon in this disease.^46^,^59^ Confocal reflectance imaging might not substitute for angiography, but the combination may improve the diagnostic sensitivity. Abnormalities of macular pigment distribution and Müller cell pathology have been suggested to contribute to the phenomenon of increased CBR.^46^,^59^

In fact, a central depletion of macular pigment has recently been established in patients with IJFT IIA.^60^ The spatial distribution of macular pigment is objectively mapped using subtraction of two fundus autofluorescence (FAF) images acquired with the modified cSLO at two different excitation wavelengths (488 and 514 nm).^60^,^61^ In IJFT IIA, the distribution of macular pigment optical density (MPOD) is reported to show a consistent abnormal pattern: a marked oval-shaped depletion of macular pigment within the central retina that corresponds well to the late-phase hyperfluorescent areas on FA, with a surrounding ring of preserved MPOD at about 6° eccentricity.^46^,^59^,^60^,^62^,^63^ This pattern represents a novel phenotypic characteristic of IJFT IIA, not seen in any retinal disease.^60^ The outer ring signal may be due to simply preserved, relatively elevated, or displaced macular pigment. This remains to be determined. Recording of macular pigment distribution may prove useful in the diagnosis of IJFT IIA. The distribution implicates an impaired trafficking or storage of lutein and zeaxanthin in this disease.^60^ Patients with IJFT IIA have shown a disproportionally high zeaxanthin reduction in one study.^62^

In addition to mapping MPOD, FAF imaging may provide the earliest fundus alterations in IJFT IIA, i.e., before angiographic signs are visible, making this technique useful for the diagnosis and monitoring disease progression.^46^,^63^ Recently, Wong *et al*.^63^ examined 22 eyes with IJFT IIA with multiple imaging methods [fundus photography, FA, OCT, FAF, and microperimetry (MP)] and proposed a classification of IJFT IIA into five categories (0–4) based on the sequence of progressive changes observed [Table 3].^63^ The authors observed that mild increases in FAF at the fovea (attributed to a loss of macular pigment) are detectable before clinical or angiographic findings are visible. As the disease progresses, foveal FAF signal becomes progressively more prominent until hyperpigmentation develops, where the FAF signal becomes mixed, showing both increased and decreased autofluorescence. This mixed FAF signal is proposed to relate to a changing composition of fluorophores in the RPE, which alludes to the possible involvement of the RPE in disease pathogenesis.^63^

**Table 3:** Categorization of IJFT IIA eyes based on combined fundus photography, FA, FAF, OCT, and MP (Wong *et al*.)^63^

Category no.	Description
0	Normal results on all imaging methods (fellow eyes)
1	Mild increased foveal autofluorescence on FAF; no other abnormalities
2	Mild-to-moderate increased foveal autofluorescence + funduscopic and angiographic features of IJFT IIA. No atrophic or cystic abnormalities on OCT imaging. No MP deficits
3	Moderate to marked increased foveal autofluorescence + funduscopic and angiographic features of IJFT IIA + foveal atrophy and cysts on OCT + centrally decreased retinal sensitivity on MP
4	Mixed patterns of increased and decreased FAF signal + clinically evident pigment clumping + central outer retinal atrophy on OCT + scotomas on MP correlating with decreased FAF signal or retinal atrophy on OCT

FA - Fluorescein angiography; FAF - Fundus autofluorescence; OCT - Optical coherence tomography; MP - Microperimetry

Wong *et al*.^63^ added three important observations:

1. Areas with mildly or moderately elevated FAF changes correlated with intact retinal structure on OCT and function on MP, whereas areas of significantly elevated or decreased FAF abnormalities correlated with disrupted retinal structure on OCT and decreased sensitivity on MP testing. These observations were corroborated by other authors.^64^
2. The five categories described did not follow a strict pattern of decreasing central VA. Similar to observations by Charbel Issa,^65^ Wong noted that VA depended less on the presence of retinal atrophy, pigment migration or hypofluorescence signals *per se*, and more on their position relative to the foveal center. Central VA is decreased when these changes occur in the fovea, but may be preserved if they are limited to the parafovea.
3. The development of SRNV was also not limited to eyes in a single category. SRNV occurred in eyes in which there was no retinal atrophy and only mild changes in FAF signal (Category 2), as well as in eyes with advanced pigment clumping (Category 4).

Again, all these observations suggest the possibility that the anatomic changes that cause the FAF alterations may precede the more typical vascular changes or atrophic changes seen in this condition.

### Functional deficits

Since the macular abnormality in IJFT IIA is mainly located parafoveally, VA testing which is dependent on foveal function, may not represent the optimal method to evaluate macular dysfunction in these patients.

IJFT IIA may lead to a sharply demarcated parafoveal scotoma that correspond topographically to the angiographically and ophthalmoscopically visible alterations and to outer retinal atrophy on OCT.^65^ These distinct parafoveal functional deficits may be present even in nonproliferative stages. Early in the course of the disease, light increment sensitivity (LIS) could be preserved despite angiographic leakage. LIS reduction then starts temporal to the fovea, progresses above and below the fovea, and involves the nasal area in later disease stages. These findings suggest that angiographic leakage precedes functional consequences, and only chronic retinal alterations may lead to a reduction of LIS.^65^ Light increment sensitivity reduction temporal to the fovea may be present despite preserved VA, but lead to the significant functional deficits and reading difficulty reported in these patients.^65^

In a prospective-controlled observational study of 49 eyes of 26 patients with IJFT IIA, reading speed and acuity were considerably reduced and correlated with parafoveal retinal LIS.^66^ Visual acuity did not significantly impact reading performance. Similarly, Schmidz *et al*.^67^ found severe photopic and scotopic scotomata next to fixation using fine matrix mapping (FMM). The authors demonstrated that IJFT IIA causes slowly progressive visual loss, in which the scotopic scotoma (rod dysfunction) occurs earlier and is larger and deeper than the photopic scotoma (cone dysfunction). Progression of scotoma correlated with enhanced late-phase hyperfluorescence and enlargement of pigmented plaques.^68^ Interestingly, severe reduction in retinal sensitivity was not spatially confined to angiographically visible alterations, but to morphological alterations seen with CBR and FAF.^67^ The findings imply, again, that IJFT IIA is not solely a vascular disease and that early neuronal involvement may be implicated in its pathogenesis.

The MacTel Study Group studied the correlation between macular thickness determined by OCT with microperimetric LIS and VA in patients with IJFT IIA and observed that median central LIS was significantly lower in eyes with normal central foveal thickness compared with eyes with subnormal central foveal thickness.^53^ Their study confirms previously described findings that the central retinal thickness is slightly lower in IJFT IIA compared with normative data, and supports the hypothesis that early stages, with still-preserved photoreceptors and retinal function, show retinal thinning before a superimposed edema leads to a slight neurosensory thickening.^47^,^53^ Later, after functional deterioration has evolved, the atrophic changes may be the predominant feature.

Metamorphopsia is a frequent symptom in IJFT IIA. In one study, it was present in 83% of eyes with nonproliferative IJFT IIA and in all four eyes with SRNV. The degree of distortion was often but not necessarily correlated with the degree of leakage.^69^

In summary, IJFT IIA has significant impact on visual functioning, even when VA is only modestly impaired. Patients with IJFT IIA reported markedly reduced visual functioning on the NEI-VFQ-25 questionnaire, and ranked among the lowest scores across other cohorts with various eye diseases including AMD and diabetic retinopathy.^70^

### Treatment

When considering treatment, we must distinguish between the therapeutic attempts for nonproliferative IJFT IIA, and treatment modalities for the SRNV of the proliferative stage.

#### Treatment of nonproliferative IJFT IIA

The angiographic late intraretinal staining pattern in IJFT IIA has prompted many ophthalmologists to interpret it as macular edema secondary to retinal vascular leakage. Several treatment modalities have been tried to treat this “macular edema.”

To start, argon laser photocoagulation (ALPC) is not effective in the treatment of nonproliferative IJFT IIA.^2^,^21^ Gass and Blodi noted that the long-term prognosis for patients with IJFT IIA is poor and laser treatment resulted in either worsened or no change in VA. Gass did not recommend ALPC in nonproliferative IJFT.^2^ Park *et al*. also found no improvement or stabilization of vision with grid ALPC. In addition, treatment was associated with retinal pigment epithelial changes; post-treatment retinal hemorrhages, vascularized retinal scars, and increased retinal vascular distortion.^21^ Laser treatment may cause the latter by stimulating fibrous tissue present in the middle or inner retina to undergo further contraction.^21^ Given the histopathologic features of IJFT IIA, where the retinal edema in the inner layers is primarily intercellular and intracellular,^24^ and where the loss of vision is primarily due to foveal atrophy rather than exudation, it is not surprising that ALPC has hardly a role in the treatment of nonproliferative IJFT IIA.

Verteporfin photodynamic therapy (PDT) was also tried in two patients with nonproliferative IJFT IIA in an attempt to reduce the permeability of the telangiectatic vessels.^71^ However, PDT improved neither vision nor the macular edema and appeared therefore not effective. No adverse effects were reported.

Preliminary case reports (five cases) of IVTA in nonproliferative IJFT IIA seemed to have an effect, with mild improvement of visual acuity, but the follow-up was very short.^72^–^74^ The retrospective study of 19 eyes by Wu *et al*., however, showed that IVTA at a dose of 4 mg does not improve VA in most eyes with nonproliferative IJFT IIA. There were no OCT controls to evaluate the effect of IVTA on retinal thickness in this study.^75^ Given that (1) UHR–OCT shows that the fluorescein leakage seen is not associated with retinal thickening,^7^ (2) the angiographic “leakage” is probably due to the staining of the extracellular matrix rather than extracellular leakage,^27^ and (3) VA correlates with photoreceptor layer disruption and not the degree of “leakage,”^7^ IVTA is likely to have a minor or no therapeutic effect in nonproliferative IJFT IIA, which coupled with a high incidence of cataract and increased intraocular pressure, suggest that it should be avoided as treatment of this condition.

Recent publications on intravitreal anti-VEGF injections, namely bevacizumab, report on possible short-term VA increase in some cases of IJFT IIA.^54^,^76^ In one case, with initial VA 20/50, treated with an intravitreal injection of bevacizumab (1.25 mg), VA improved but macular edema recurred after 3 months, requiring a repeat injection with no significant improvement of VA thereafter.^76^ Another retrospective series of seven eyes treated with two doses of intravitreal bevacizumab (1.5 mg) at 4-week intervals demonstrated a short-term decrease in retinal thickness and a reduction in angiographic leakage.^54^ When these same patients received further injections, depending on disease activity, and were followed for approximately 18 months, it was noted that mean VA had increased, central retinal thickness had decreased following each treatment, but treatment effect appeared less pronounced with subsequent injections, and rebound effect recurred earlier than it had after the initial two injections. At the last visit, retinal thickness had increased in selected retinal sectors including the fellow eye.^77^ The authors concluded that intravitreal bevacizumab for nonproliferative IJFT IIA has only a transient effect.^77^ The authors nevertheless proposed that inhibition of VEGF may be useful particularly before atrophic changes occur. They believed that VEGF plays a pathophysiological role in IJFT IIA, because, in their opinion, the structural capillary changes described histopathologically lead to a disturbed exchange of oxygen and substrates between the vascular lumen and neurosensory retina, which in turn may lead to a hypoxia-induced increased VEGF release by retinal cells.^77^

However, in another two patients with nonproliferative IJFT IIA treated with intravitreal bevacizumab (1.25 mg) and followed for 12 months, leakage on FA decreased likewise, underlining the effect of bevacizumab on vessel stability and permeability, but this was not accompanied by an increase in VA despite triple injections.^78^ Furthermore, small cystic changes seen on OCT remained unchanged, emphasizing that visual deterioration is caused by microcystic degeneration and progressive retinal atrophy and not by intraretinal edema, and therefore cannot be halted with intravitreal anti-VEGF injections. A more recent and larger retrospective review of nine eyes treated with intravitreal bevacizumab and followed from 4 to 27 months, corroborated that intravitreal bevacizumab decreased FA leakage but had no short-term effect on VA or OCT appearance.^79^ Similar results to these two reports were observed with intravitreal injections of pegaptanib.^80^ These three studies suggest that eyes with minimal cystic changes on OCT do not show functional improvement despite repeated intravitreal anti-VEGF injections,^78^,^80^ and that there seems to be no apparent visual acuity or OCT benefit to using intravitreal anti-VEGF in the absence of SRNV.^78^,^79^ Moreover, it has been suggested that VEGF plays a role in photoreceptor differentiation, may contribute to photoreceptor survival, and may serve a role in maintaining retinal vascular homeostasis.^81^ Therefore, it cannot be ruled out that blocking VEGF may cause an acceleration of apoptosis among ganglion cells and photoreceptors in IJFT IIA.^77^ This remains to be tested.

In summary, it is unclear yet if anti-VEGF injections would be of value in the treatment of nonproliferative IJFT IIA. Given the current lack of convincing evidence of efficacy, the concern about the potential deleterious effects of repeated injections, including endophthalmitis, and cost of treatment, continued therapy of nonproliferative IJFT IIA with VEGF antagonists appears, at this time, questionable.

#### Treatment of proliferative IJFT IIA

The natural history of untreated SRNV in IJFT IIA is generally poor^33^,^43^ with 80% of eyes in a series of 26 eyes having a final VA of 20/200 or worse.^33^ Before the advent of VEGF antagonists, therapeutic options for SRNV associated with IJFT IIA included laser photocoagulation, PDT with or without IVTA, transpupillary thermotherapy (TTT), and surgical removal of the SRNV.^22^,^82^–^87^

Ablation of the SRNV by photocoagulation has been described as being able to prevent deterioration of VA only in selected cases.^2^,^33^ This treatment, however, is only feasible if the SRNV is distant enough from the foveal center. Prognosis for visual recovery is guarded despite laser,^2^ and treatment will result in a scotoma close to fixation.

Verteporfin PDT was reported to produce SRNV obliteration in IJFT IIA but with modest visual results.^82^,^83^,^88^ In one case treated with PDT reported by Potter *et al*., visual improvement of one line was achieved. Leakage from the SRNV on FA was halted, but leakage from the juxtafoveal telangiectasis continued.^82^ However, in the four cases treated by Snyers *et al*., three maintained baseline vision while one eye had further deterioration of vision even after multiple sessions of PDT.^83^ An average of 2.4 PDT treatments were required in one series for the cessation of leakage.^88^ Shanmugam *et al*. reported one case in which SRNV showed partial regression after PDT, however retinal pigment epithelial atrophy corresponding to the size of the laser spot developed.^89^ Similar RPE collateral damage was noted in another series, but vision remained stable.^90^ It is postulated that the photosensitizing agent may leak out of the retinal vessels and cause collateral damage to the macula when activated.^89^,^90^ The risk of permanent RPE damage raises a doubt regarding the safety of PDT for SRNV in IJFT IIA. Combined PDT and IVTA of 4 mg has shown in one case cessation of leakage after two treatment cycles and a 6-line visual improvement at 9 months of follow-up.^91^

Transpupillary thermotherapy using low power (150–500 mW, mean 325 mW) has also been reported to be effective in stabilizing or improving the visual function in 12 out of 13 eyes with SRNV secondary to IJFT IIA.^86^ Another series reported encouraging results in nine eyes, treated with TTT, mostly with a single application, with 88% eyes maintaining or achieving better vision at a mean 9.7-month follow-up.^87^ Transpupillary thermotherapy does not have any effect on the telangiectasia, and angiographic leakage from the telangiectatic vessels persisted in most of the cases, despite SRNV regression.

Surgical removal of subfoveal SRNV was attempted by Berger *et al*. in two patients with IJFT IIA resulting in poor postoperative visual outcome.^22^ The removal is difficult to perform because of intimate adherence of the SRNV with the overlying neurosensory retina in the area of retinochoroidal anastomosis. Submacular surgery is, therefore, not considered beneficial in this condition.

Vascular endothelial growth factor has been implicated as the major angiogenic stimulus responsible for neovascularization in IJFT IIA. Given the risk of permanent RPE damage with PDT, coupled with the huge evidence of efficacy of VEGF antagonists in the treatment of choroidal neovascularization in various entities, the anti-VEGF approach is a reasonable treatment alternative for proliferative IJFT IIA, particularly in the presence of retinochoroidal anastomosis. Several case series in the literature investigated bevacizumab or ranibizumab for the treatment of proliferative IJFT IIA but with limited (12 months or less) follow-up.^92^–^99^ In a nonrandomized, interventional case series of six eyes with proliferative IJFT IIA that received a single injection of intravitreal bevacizumab (1.25 mg), VA improved two or more lines in five eyes (83%) and remained the same in one eye (17%) at final follow-up (3–6 months). Mean central foveal thickness decreased by 62 μm. All eyes demonstrated reduction in the subretinal fluid, intraretinal edema, associated hemorrhage, and angiographic leakage of both the SRNV and telangiectasis.^92^ Several case reports of treatment with one or more intravitreal injections of bevacizumab (1.25 mg)^93^,^94^,^96^ or ranibizumab (0.5 mg)^95^,^97^,^98^ injections with a mean 6 months follow-up, corroborated the improvement in VA, the resolution of subretinal and intraretinal fluid on OCT and the regression of SRNV with cessation of SRNV leakage in all cases. Leakage from the telangiectasis also decreased, but recurred by six months.^94^,^97^ No obvious adverse events were noted. In a more recent retrospective series of five eyes with proliferative IJFT IIA treated with intravitreal bevacizumab with longer follow-up (4–27 months), best-corrected VA was unchanged or improved after treatment. All eyes demonstrated decreased intraretinal leakage and decreased growth and leakage of the SRNV. As expected in IJFT IIA, the decrease in central retinal thickness on OCT was the modest (mean < 30 μm).^79^

Primary treatment with combined intravitreal injections of bevacizumab (1.25 mg) or ranibizumab (0.5 mg) and PDT were also reported anecdotally for proliferative IJFT IIA.^100^,^101^ In both cases, the PDT was performed with a laser spot of the same size as the SRNV and followed by the intravitreal injection. In one case, which required repeated injection of bevacizumab, VA had improved by 16 months from 20/600 to 20/200 and the SRNV regressed and was scarred.^100^ In the second case treated with ranibizumab and PDT, VA improved by two lines at 16 weeks and SRNV regressed clinically, angiographically, and on OCT. The results were stable throughout the follow-up of 9 months. No adverse effects were noted.^101^

In conclusion, current preliminary results suggest that intravitreal delivery of anti-VEGF therapy combined with or without PDT appears efficacious and should be considered as a treatment option for proliferative IJFT IIA. Long-term follow-up and larger series are needed to address the long-term outcomes, the needed frequency of anti-VEGF drug delivery, and specific side effects or complications of anti-VEGF therapy in this condition.

### Differential diagnosis

Foveolar atrophy in IJFT IIA may simulate a macular hole. OCT permits the distinction. Some eyes demonstrate in the early stages a yellow foveal lesion that may be mistaken for adult vitelliform dystrophy or Best’s disease.^27^ Retinal crystals may be mistaken for other causes of crystalline retinopathies, but FA readily establishes the correct diagnosis. The macular pigment plaques with SRNV may be mistaken for age-related macular degeneration, but drusen and pigment epithelial detachment are generally absent in IJFT IIA.

## GROUP III: OCCLUSIVE IDIOPATHIC JUXTAFOVEOLAR RETINAL TELANGIECTASIS

**Figure 10:**
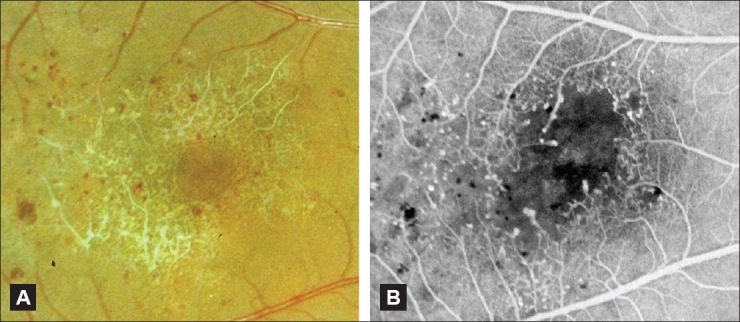
(A and B) Color fundus photograph (A) and corresponding fluorescein angiogram (B) of an eye with occlusive IJFT (group III). Note the marked perifoveolar capillary nonperfusion seen clinically and angiographically. There are numerous telangiectatic vessels as well that cause limited exudation along the vascular walls. Limited exudation in IJFT III was described by Gass and Blodi[2]

This is a rare form of IJFT described by Gass characterized by progressive bilateral perifoveolar capillary obliteration, capillary telangiectasis, minimal exudation clinically and on FA and visual loss in association with systemic or cerebral familial disease [Table 1][Figure 10A and B].^1^,^2^ It has been omitted in the recent classification of IJFT based on its rarity.^3^

## CONCLUSION

Idiopathic juxtafoveolar retinal telangiectasis comprises essentially three groups that are that considerably different in their appearance, their presumed pathogenesis and management strategies. In group I, the unilateral telangiectasis is easily visible and vision loss is primarily a result of serous and lipid exudation in the macula. Photocoagulation is generally effective in controlling the macular edema. In group II, the most common, the bilateral capillary telangiectasis is more difficult to detect biomicroscopically, but the angiographic and OCT findings are characteristic and diagnostic. Vision loss is progressive and primarily due to retinal atrophy, not exudation. More rapid visual loss can occur with SRNV, not uncommonly. Treatment options for this group are still very limited, and have shown effectiveness only for the subretinal neovascular component. This is primarily because the pathogenesis of this telangiectasis remains an enigma and is possibly secondary to a retinal neuronal dysfunction. New imaging modalities and functional tests, along with worldwide longitudinal studies will hopefully improve the understanding and treatment capabilities of this condition. As for group III, it is featured primarily as a perifoveolar capillary occlusive condition, and is poorly understood because of the scarcity of cases reported.
